# Impact of silver nanoparticles on secondary metabolite composition and toxicity in anise (*Pimpinella anisum L.*) callus culture

**DOI:** 10.1186/s12870-024-05067-8

**Published:** 2024-05-04

**Authors:** Esma Ulusoy, Aysenur Bozkurt, Sumeyye Durmaz, Huseyin Servi, Filiz Vardar, Semiha Erisen

**Affiliations:** 1 Department of Molecular Biology and Genetics, Faculty of Engineering and Natural Sciences, Uskudar University, P. O. Box 34662, Istanbul, Turkey; 2 Department of Pharmacy, Faculty of Pharmacy, Yeni Yüzyıl University, Istanbul, Turkey; 3 Department of Biology, Faculty of Science, Marmara University, Istanbul, Turkey; 4 Department of Molecular Biology and Genetics, Faculty of Science and Letters, Yildiz Technical University, Istanbul, Turkey

**Keywords:** AgNP, Secondary metabolites, Anise, Toxicity, *Pimpinella anisum*, *Fatty* acids

## Abstract

**Background:**

There are numerous challenges associated with producing desired amounts of secondary metabolites (SMs), which are mostly unique and cannot be chemically synthesized. Many studies indicate that nanoparticles (NPs) can boost the production of SMs. Still, the precise manner in which NPs induce metabolic changes remains unidentified. This study examines the influence of eco-friendly silver NPs (AgNPs) on the chemical makeup and toxicity of *Pimpinella anisum L.* (anise).

**Results:**

AgNPs were introduced into anise callus cultures at different concentrations (0, 1.0, 5.0, 10, and 20 mg/L). The induced oxidative stress was tracked over intervals of 7, 14, 28, and 35 days. Chemical composition evaluations were carried out on the 35th day. Within the first 14 days, plant stress was evident, though the plant adapted to the stress later on. Notably, the plant showed high tolerance at 1 mg/L and 5 mg/L concentrations despite increased toxicity levels. However, relatively high toxicity levels were identified at 10 and 20 mg/L. The AgNP-induced stress significantly impacted anise SMs, particularly affecting fatty acid content. In the 10 and 20 mg/L AgNP groups, essential metabolites, including palmitic and linoleic acid, showed a significant increase. Polyunsaturated (omega) and monounsaturated fatty acids, vital for the food and pharmaceutical industries, saw substantial growth in the 1 and 5 mg/L AgNP groups. For the first time, vanillyl alcohol and 4-Hydroxybenzoic acid were detected along with various phenolic compounds, such as t-anethole, Salicylic acid, and Thiamazole.

**Conclusion:**

AgNPs can function as an elicitor to efficiently generate essential SMs such as omegas and phenolic compounds in anise callus culture. This study explores the application of AgNPs as plant elicitors in anise SM production, offering invaluable insight into potential uses.

## Introduction

The negative side effects of synthetic drugs derived from plants have sparked increased interest in therapies based on plant extracts in recent years. Plants are generally more resistant to adaptation than widespread species. Conversely, endemic species evolve unique traits to survive and adapt to microclimates, often inhospitable to many other species. Certain studies suggest that these traits can also influence the survival of other plants in the ecosystem [1]. Research indicates that plants produce chemical molecules known as SMs. These SMs offer various benefits in their ecosystem interactions, environmental adaptation, defense, and survival. They are also economically valuable in sectors like pharmaceuticals, chemistry, food, cosmetics, and agriculture [2]. Most plant SMs are unique to a particular plant genus or even a single species and, therefore, cannot be replicated by other plants [3].

*Pimpinella anisum L.*, part of the Apiaceae family, is an aromatic plant famed for its varied historical uses [4]. Anise plants can be found in locations such as Turkey, Egypt, Iran, Iraq, India, Latin America, South Africa, and many temperate regions, particularly in the Eastern Mediterranean and Western Asia [5]. The primary exporters are Turkey, Egypt, and Spain [6]. There are 37 species of the *Pimpinella* genus native to Turkey, with nine being endemic. *P. anisum L.*, commonly known as anise, is an annual herbaceous plant, approximately 30–50 cm in height, boasting white flowers and small green/yellow seeds [7].

Turkey enjoys a particularly diverse array of plant species (nearly 12,000). This diversity suggests a rich range of SMs produced by these plants. In 2010, 14,000 tons of aniseed was harvested in Turkey, which increased to around 18,000 tons in 2019. Approximately 2,000 to 4,000 tons are exported each year, making Turkey the sixth largest global producer, contributing 2.3% of the world’s aniseed [8]. Local growers manage the majority of anise production, as commercial certified seeds are not available. The seeds used come from a variety of populations, and the yield and anethole content can vary depending on the genotype, ecological conditions, and specific farming practices, including irrigation, manuring, and sowing dates [9]. Therefore, inconsistency is an issue in the anise industry, often affecting the standard of the final product. Tissue and cell culture methods are explored as potential solutions to these challenges, promising to deliver the desired quality and quantity of SMs.

The dried fruits of anise, known as anise seeds, possess a distinct, enticing aroma. Thus, these are a popular flavoring addition in everything from candy to liqueurs to other culinary creations [9]. Anise is primarily recognized for its beneficial impact on digestive issues, including flatulence, indigestion, and colic. Additionally, the seeds are believed to boast carminative and expectorant abilities. Given its antimicrobial, antioxidant, and anti-inflammatory effects, anise holds significance in natural medicine [10]. Its analgesic and anticonvulsant effects indicate its potential for managing pain and treating neurological disorders characterized by seizures [11, 12]. Research suggests that anise’s essential oil extract can lessen symptoms of polycystic ovary syndrome and influence luteinizing hormone levels. Consuming anise oil may alter testosterone levels and affect sperm count and motility [13]. Moreover, anise has demonstrated antidepressant activity comparable to fluoxetine, suggesting it might hold substantial clinical relevance in depression management [14].

Arslan et al. [5] conducted research on Turkish anise, discovering that the ratios of its essential oil varied from 1.3 to 3.7% across population samples. The proportion of trans-anethol, the primary component of this essential oil, ranged between 78.63 and 95.21% and 1.5–6.0% by mass. Anise seed’s secondary essential oil contains several vital compounds, such as eugenol, methyl chavicol, anisaldehyde, estragole, umbelliferon, sterols, terpene hydrocarbons, polyenes, and polyacetylenes [15]. It also featured significant amounts of γ-heachalene, p-anisaldehyde, methylchavicol, cis-anethole, α-cuparene, α-heachalene, β-bisabolene, (E)-methylugenol, gallic acid, rosmarinic acid, and phenolic acid varieties [16]. Other components included lipids, accounting for 8–11% fatty acids like oleic and palmitic acids, and flavonoids like coumarin, flavanol, rutin, flavone, isoorientin, glycosides, and isovitexin [17, 18], as well as betaarmyrin, and stigmasterol. Finally, anise comprises approximately 4% carbohydrates and 18% protein [19].

Early studies detected significant components, sesquiterpene hydrocarbons and anethole, in callus cultures derived from roots and leaves due to alterations in the auxin/cytokinin balance in the culture medium [20]. Comparative studies revealed varying concentrations of anise’s SM content across different plant parts, callus, and cell suspension cultures: anethole content was 92% in fruit, 50% in the shoot, 4.9% in root and 17% in callus. Additionally, compounds such as epoxypseudo-isougenol-2-methylbutyrate, p-bisabolene, and myristicin were detected in the callus but were absent in the fruit, shoot, or root [21].

More recent research demonstrated an increase in t-anethole content in anise callus cultures exposed to salt stress, measuring 77.09 ppm in callus, 78.42 ppm in seed, and 27.77 ppm in leaf [22]. Similar outcomes were observed under water stress conditions [23]. Another study showed that in vitro cultures of anise treated with methyl jasmonate, an elicitor, stimulated phenolic acid metabolism and increased chromium (eugenin) accumulation [24]. Collectively, these studies indicate that the SM content of anise in vitro cultures is affected by stress and elicitor applications.

Numerous studies have shown that nanoparticles (NPs) impact plant physiology, growth, and development [25–27]. For instance, low concentrations of silver NPs (AgNPs) have been found to enhance the growth of shoots and roots in many species [26]. Nevertheless, research on the effects of NPs on the production of plant SMs in in vitro cultures is lacking [28]. The few existing studies indicate that applying 900 mg/L AgNPs to *Artemisia annua L.* fringe root cultures saw a 3.9-fold increase in artemisinin content over 20 days [29]. Additionally, an increase in both plant growth and diosgenin concentration was observed in fenugreek after exposure to 2 µg/kg AgNPs [30]. Barley plants treated with cadmium oxide NP for 3 weeks showed increased ferulic acid and isovitexin content [31]. Alongside these positive results, the potential toxicity of NPs has also been reported, occurring in a concentration-dependent or independent fashion. Given the ability of in vitro methods to significantly alter SM content via adjustments to nutrient medium and stress conditions [32], these techniques might enable the production of SMs in the desired quality and quantity.

NPs can alter gene expression across various pathways, including those related to biotic/abiotic stress responses, cell development, cell organization, electron transport, and energy [33, 34]. Additionally, they can induce oxidative stress [35]. It has been hypothesized that NPs, acting as abiotic stress factors, may affect SM mechanisms, with some studies indicating their potential use as elicitors. For instance, nano-titanium dioxide NPs have been suggested as potential elicitors to enhance antioxidant biosynthesis and secondary metabolism in *Salvia officinalis* [32]. However, NPs can also cause toxicity in plants [36]. Different plant species might employ unique detoxification mechanisms to diminish the toxic effects of AgNPs. Thus, concluding how these disparate detox pathways are activated in response to different AgNP conditions across different plant species can be complex [26, 27, 37]. Assessing these differential responses highlights the influence of properties such as AgNP size, shape, exposure concentration, surface coating, silver form, and aggregation state on each plant’s morphology, physiology, and biochemistry [26, 38].

Regarding anise, a valuable agricultural crop, studying the impacts of differing elicitors is critical. Literature reviews suggest that factors such as type, quantity, and plant species significantly affect NP penetration into cells. Therefore, exploring the impact of each elicitor on anise is a necessary research direction.

Moreover, the nanoparticle synthesis method is crucial. Chemical methods often result in toxic solvents, significant energy expenditure, and harmful byproduct formation [39]. Thus, we employed an eco-friendly green synthesis method for AgNPs production. We utilized *Salvia sclarea* leaves in the synthesis, a plant we are experienced in optimizing.

**Fig. 1:**
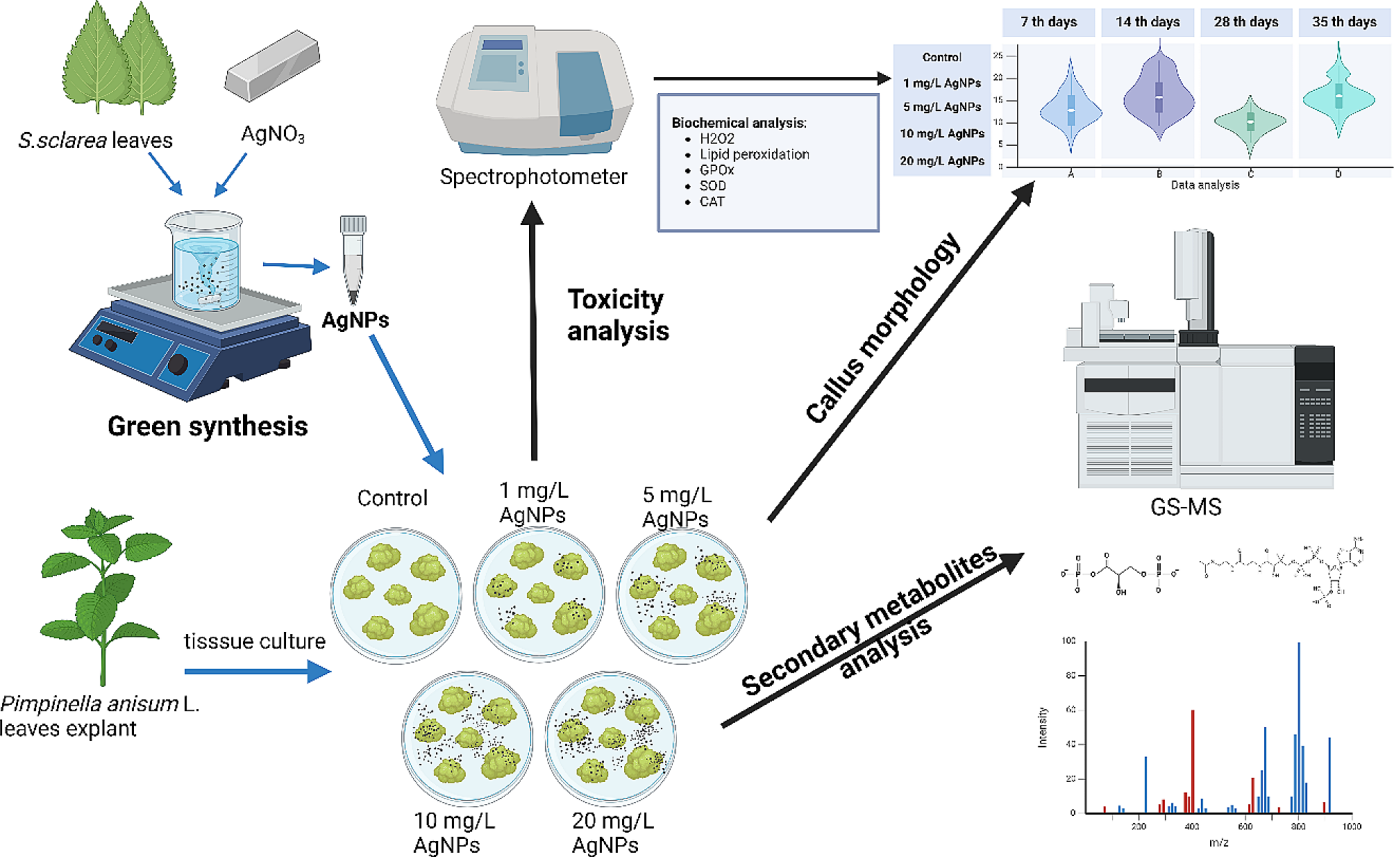
Brief summary of the study

We conducted our study using the anise plant from the Burdur region, which was noted for its high secondary content and diversity [10]. This study holds originality as it is the first to explore NP-SM interactions of anise. A summary of the study can be found in Fig. 1.

The positive findings from this study could improve the use of in vitro callus cultures. This innovation in nanobiotechnology could offer solutions to environmental issues and genotype factors that affect the anise plant’s production of valuable metabolites.

## Materials and methods

### Preparation of extracts from *Salvia sclarea* (clary sage) leaves and AgNPs green synthesis

**Fig. 2:**
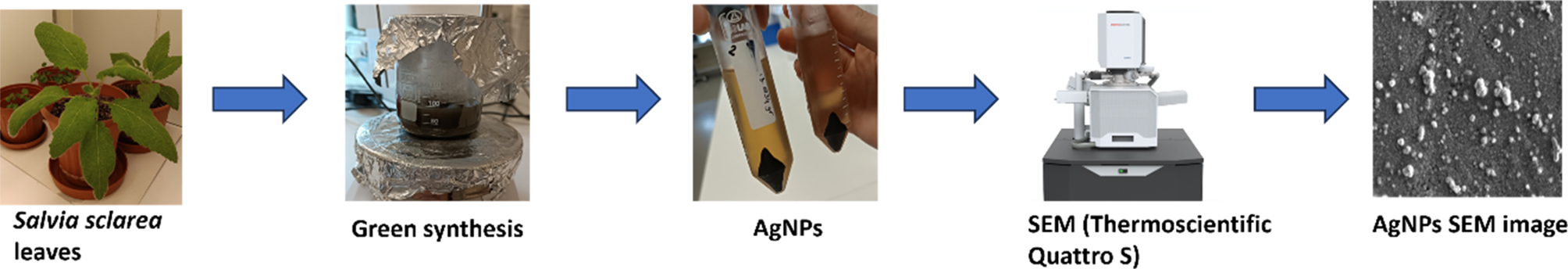
The Green Synthesis method produced AgNP with *S. sclarea* leaf extracts

The production of AgNP was conducted through the green synthesis method, employing *S. sclarea* leaf extracts (Fig. 2). The clary sage leaves were sourced from the Tissue Culture Laboratory in the Department of Molecular Biology and Genetics at Yıldız Technical University (YTU).

First, the leaves were washed, chopped, and dried on filter paper. They were then boiled in distilled water for 30 min. The extract was filtered and centrifuged at 6000 g for 10 min to clear out any lingering particles. A green synthesis of AgNPs then occurred using Salvia Leaf Extract, which involved adding the extract to a 1 mM AgNO_3_ (silver nitrate) solution (Sigma-Aldrich-7761-88-8, St. Louis, Missouri, USA) at a ratio of 1:9. To optimize the process, the pH was increased from 5 to 6 to 9, and the mixture was incubated in a magnetic stirrer at 50–60 °C for 30 min. Observable color changes marked the formation of AgNPs. The NPs were separated using a centrifuge (Hermle Z383 K, Germany) at 10,000 g for 1 h, then lyophilized (Telstar Cryodos-50, Spain) and converted into powder.

### **Characterization of AgNPs via spectroscopy and scanning electron microscopy** (**SEM**)

The start of AgNP formation was marked by a change in color from light yellow to dark brown during incubation [40]. The finalized AgNPs were examined through suitable spectral analyses. The AgNP solution’s absorption spectra were logged by a UV-Vis spectrophotometer (ThermoScientific-Genesys 180 UV-v Spectrophotometer) across a range of 300 to 600 nm. Peaks appearing from 400 to 467 nm were taken as the AgNP wavelength [40, 41]. Particle size was quantified at the Department of Bioengineering at YTU using a Zetasizer spectroscopy device (Malvern Zetasizer-NanoS, Cambridge, UK).

A Scanning Electron Microscope (Thermoscientific Quattro S) was employed to observe the surface morphology of the NPs at an accelerating voltage of 30 kV. After centrifugation of the AgNP solution, the precipitate was lyophilized for drying. Afterward, the dehydrated NPs were re-suspended in a liquid medium and placed on a stub (sample holder). The scanning electron imaging was carried out under a high vacuum at 2.00 kV.

### Cultivation of aseptic callus from suspension cultures

The study utilized seeds procured from Durmuş EFE, an anise grower in Kozagac village, located in the Burdur region. The research was conducted in the tissue culture labs of YTU. The seeds underwent surface sterilization by immersing them in 20% sodium hypochlorite for 5 min with the help of a magnetic stirrer. Post sterilization, the seeds were washed with sterile distilled water for 5 min, and this rinse cycle was repeated thrice. Upon sterilization, the seeds were moved to Murashige and Skoog (MS) medium [42] for additional growth. It took one week for the anise seeds to germinate in the MS medium.

**Fig. 3:**
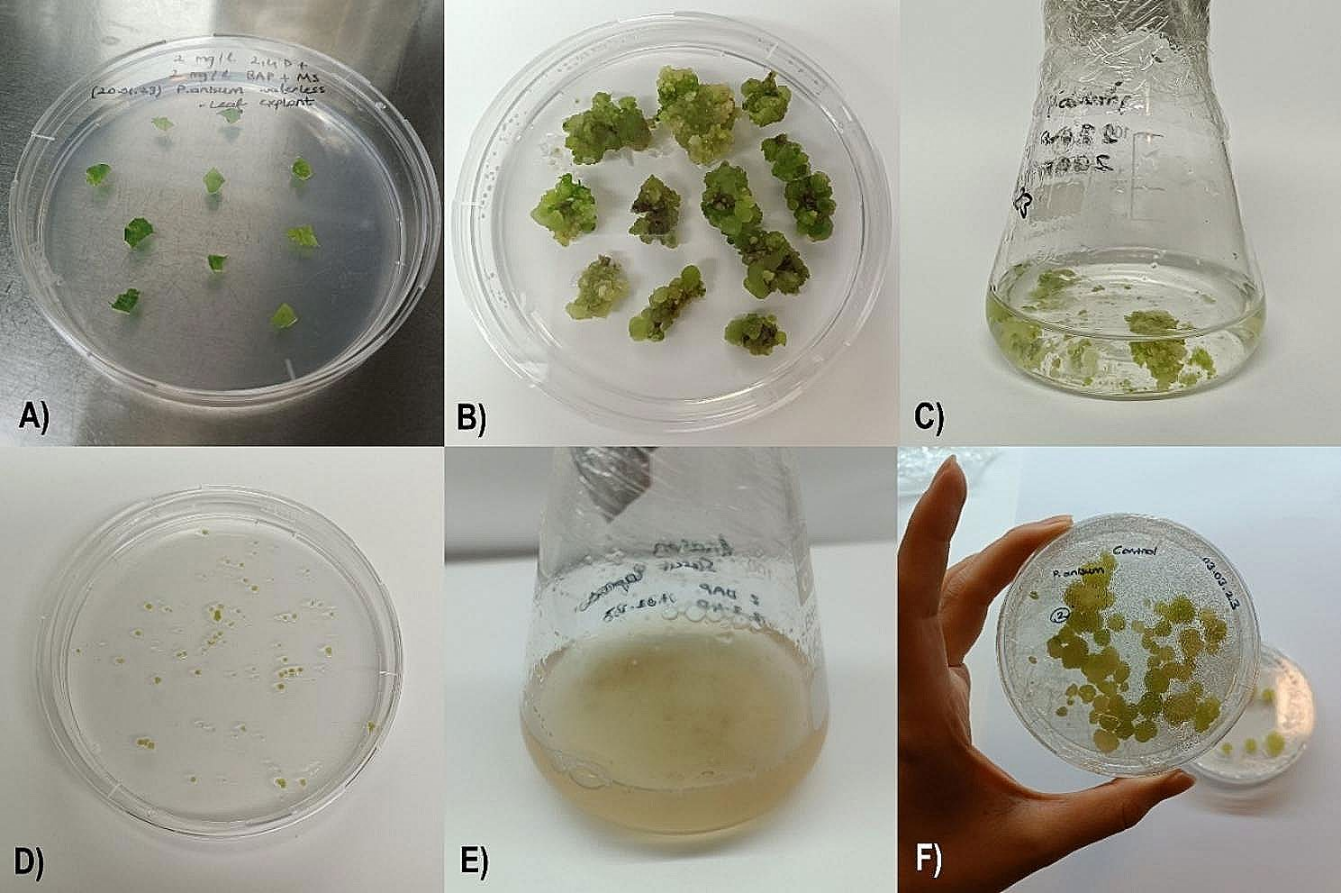
(**A**) Preparation of anise leaf explant. (**B**) A callus of an anise leaf was developed within 21 days. (**C**) A suspension medium, MS containing 2 mg/L BA and 2 mg/L 2,4-D, was used to start the suspension calli. (**D**) The anise suspension culture has been subcultured after three weeks. (**E**) After transferring the growing callus from subcultures to MS, a solid nutrient medium containing 2 mg/L BA + 2 mg/L 2,4-D, (**F**) was developed

Leaf parts from sterile anise seedlings, 3–4 weeks after germination, were used as explants. The explants were then placed in an MS medium enhanced with 2 mg/L of 2,4 D. After 3 weeks, 1 g of the resulting calli was transferred to an MS suspension medium [22] containing 2 mg/L each of BA and 2,4-D, yielding suspension calli. The cultures were continuously agitated at 110 rpm with an orbital shaker. Subsequently, the calli were relocated to fresh medium, with the same amounts of BA and 2,4-D. After that, the callus was moved to a solid MS nutrient medium with identical concentrations of BA and 2,4-D. Ultimately, the callus matured (Fig. 3).

### Application of AgNPs to anise callus cultures

After 5 weeks, the developing callus tissues were segmented into pieces weighing up to 250 mg. These were then subcultured in a solid MS medium containing 2 mg/L BA, 2 mg/L 2,4-D hormone, and varying concentrations of AgNPs (0, 1, 5, 10, and 20 mg/L). In our study, Group 0, serving as the control group, consisted of 2 mg/L BA and 2 mg/L 2,4-D hormones in the MS medium but lacked AgNPs. The cultures were then stored in an acclimatization chamber (SANYO MLR-351 H), where they were exposed to a photoperiod of white, fluorescent light (50 µmol/m^2^/s lux) for 16 h at a temperature of 25 ± 2°C.

### **Biochemical analysis** (**non-enzymatic and enzymatic antioxidants**)

We obtained callus samples from each group after treatment to examine potential toxicity effects. Each sampling was conducted on the seventh, 14th, 28th, and 35th days. We focused our study on the levels of reactive oxygen species (ROS), which induce oxidative stress. We used non-enzymatic antioxidants, including hydrogen peroxide and lipid peroxidation, and enzymatic antioxidants, such as superoxide dismutase, guaiacol peroxidase, and catalase, to analyze the samples on specific days.

We used the protocol from Junglee et al. [43] to estimate the hydrogen peroxide (H_2_O_2_) content. We homogenized a 300 mg sample with 2 ml buffer (10 mM PBS pH 7, 0.1% TCA, KI) for 10 min at        ^+ ^4℃. The mixture was then centrifuged (Sigma 3–30 K, Germany) at 12,000 g for 15 min at ^+ ^4℃. Afterward, the supernatant was allowed to settle at room temperature for 20 min in the dark. The sample’s absorbance was spectrophotometrically measured (IMPLEN NanoPhotometer-P330, Germany) at 390 nm. The H_2_O_2_ content was determined as nMol/g by applying the formula y = 0.053x − 0.021 from the standard curve graph.

To evaluate the quantity of Malondialdehyde (MDA), a product of lipid peroxidation, a 0.2 g sample was blended in 1 ml 0.1% TCA for 10 min at ^+^ 4℃. Following this, the mixture was centrifuged at 12,000 g for 20 min. To the 250 µl of supernatant, 1 ml 20% TCA containing 0.6% TBA was added and heated to 95℃ for 30 min. Swiftly, it was cooled on ice and centrifuged again at 12,000 g for 10 min. The absorbance of the supernatant at 532 and 600 nm was recorded, and the MDA quantity was determined in nmol/L using the formula Δ (532–600)/1.56 × 10^5^ as per reference [44].

Enzymatic antioxidant assays were conducted on anise calli, following the method by Lee and Lee [45]. A mixture of 0.5 g of roots and 1 ml of extraction buffer (0.1 mM EDTA, 1% PVP, 0.5% Triton X-100, 100 mM PBS with a pH of 7.8) was homogenized at ^+^4℃. It was then centrifuged (using Sigma 3–30 K, Germany) at 18,000 g for 20 min also at ^+^4℃. The supernatant served as the basis for determining the activities of guaiacol peroxidase (GPOx), superoxide dismutase (SOD), and catalase (CAT).

The GPOx assay utilized a measurement buffer, which comprised 100 mM PBS (pH 5.8), 5 mM H_2_O_2_, and 15 mM guaiacol. Into this buffer, 10 µl of supernatant was added. Measurements were taken from 1.5 ml quartz tubes using an IMPLEN NanoPhotometer-P330 spectrophotometer from Germany. The samples were measured kinetically at 470 nm for 2 min. GPOx activity was calculated using the formula ΔAbs (470 nm)/(min × mg protein) and presented as U/mg protein [46].

For the CAT assay, 1.5 ml of measurement buffer (200 mM PBS pH 7, 71 mM H_2_O_2_) was heated to 30℃ for 3 min. This buffer was then transferred to a quartz cuvette and supplemented with 37.5 µl of supernatant. Spectrophotometric measurements were taken at 240 nm for 2 min. CAT activity was determined by applying the formula ΔAbs (240 nm)/(min × mg protein) to the reducing absorbance values. Results were conveyed as U/mg protein [47].

For the superoxide dismutase (SOD) assay, we added 2 ml of measurement buffer (comprising 100 mM PBS with pH7, 2 M Na_2_CO_3_, 0.5 M EDTA, 300 mM L-Methionine, and 7.5 mM NBT), 2 µl supernatant, and 20 µl riboflavin (at 0.2 mM). We then exposed the samples to a 15 W white fluorescent light until we noticed a color change. After this change, we took absorbance measurements at 560 nm for the samples. We determined the SOD enzyme activity using this formula: (Abs560 nm × 100)/(Positive control Abs560 × min × mg protein) and presented the results as U/mg protein [48].

### Callus morphology

Changes in callus development, fresh weight, and diameter were measured at intervals of 7, 14, 28, and 35 days following the cultivation of both control and experimental groups. This allowed for the assessment of morphological changes in the callus.

### Obtaining callus extracts by maceration

The calli in the control group and those treated with AgNP were fully dried with a lyophilizator, which removed all water from their structure. The weights of the dried calli were 2.02 g, 3.93 g, 4.06 g, 4.26 g, and 2.06 g for the control, and the 1.0 mg/L, 5.0 mg/L, 10 mg/L, and 20 mg/L AgNP groups, respectively. Hexane solvent was then added to cover them, and they were left in an ultrasonic bath for one hour. The control and 20 mg/L AgNP groups received 50 ml of hexane solvent, while the remaining groups were each given 100 ml. After ultrasonication, they were left in hexane solvent overnight. The next day, the liquid portion was filtered into an Erlenmeyer flask using filter paper. This maceration process was repeated three times, combining the liquid parts each time. The hexane solvent was then evaporated at ^+^40ºC in an evaporator, resulting in crude hexane extracts with weights of 0.03 g, 0.04 g, 0.06 g, 0.03 g, and 0.05 g, respectively. The same procedures were performed for the ethanol extracts; three rounds of 100 ml ethanol were added to each group. These were then evaporated at ^+^45ºC, producing crude ethanol extracts weighing 0.42 g, 0.38 g, 0.38 g, 0.38 g, 0.40 g, and 0.40 g, respectively.

*Trimethylsilyl (-TMS) Derivatization*: The hexane and ethanol extracts underwent derivatization prior to GC-MS analysis, which identified volatile and non-volatile components. Different derivatization methods were used for hexane and ethanol extracts.

### **Secondary metabolite content analysis** (**GC-MS analysis**)

The derivatized extracts were evaluated using an Agilent 5975 GC-MSD system on an Innowax FSC column (60 m × 0.25 mm, 0.25 μm film thickness) with helium as the mobile phase (1 mL/min). The GC oven temperature was set at ^+^60 °C for 10 min, then increased to ^+^220 °C at a rate of ^+^4 °C/min. It was then maintained at^+^220 °C for 10 min before rising again to ^+^240 °C at 1 °C/min. The injections were administered in splitless mode, with the temperature set at ^+^250 °C. An ionization energy of 70 eV was used in the mass spectrometer, which was calibrated to a scanning range of m/z 35–450 atomic mass units. The GC-MS analysis was done twice for accuracy, and the resultant data were averaged.

The compounds’ relative percentages in the hexane and ethanol extracts were determined separately by integrating the mass chromatograms’ peaks. The identification of the extract components was confirmed by comparing the relative incidence indices (RRI) and mass spectra of the n-alkane (C5-C30) series, as detailed in the literature. The comparison utilized the Wiley 8th Ed./NIST 05 Mass Spectra Library, Adams Essential Oil Mass Spectral Library, and Palisade 600k Complete Mass Spectra Library [49].

### Data analysis

The in vitro culture and AgNP treatments were conducted in triplicate. Changes in callus height and weight were examined using a Tukey multiple comparisons test with a significance level of *p* > 0.05, and the results were calculated using a two-way analysis of variance (ANOVA). Antioxidant enzyme assay data underwent a similar process, analyzed with Dunnet’s multiple comparisons test at a significance level of *p* > 0.05 and calculated using two-way ANOVA. All analyses were performed using the GraphPad Prism-10 software.

## Results

### Characterization of AgNPs

**Fig. 4:**
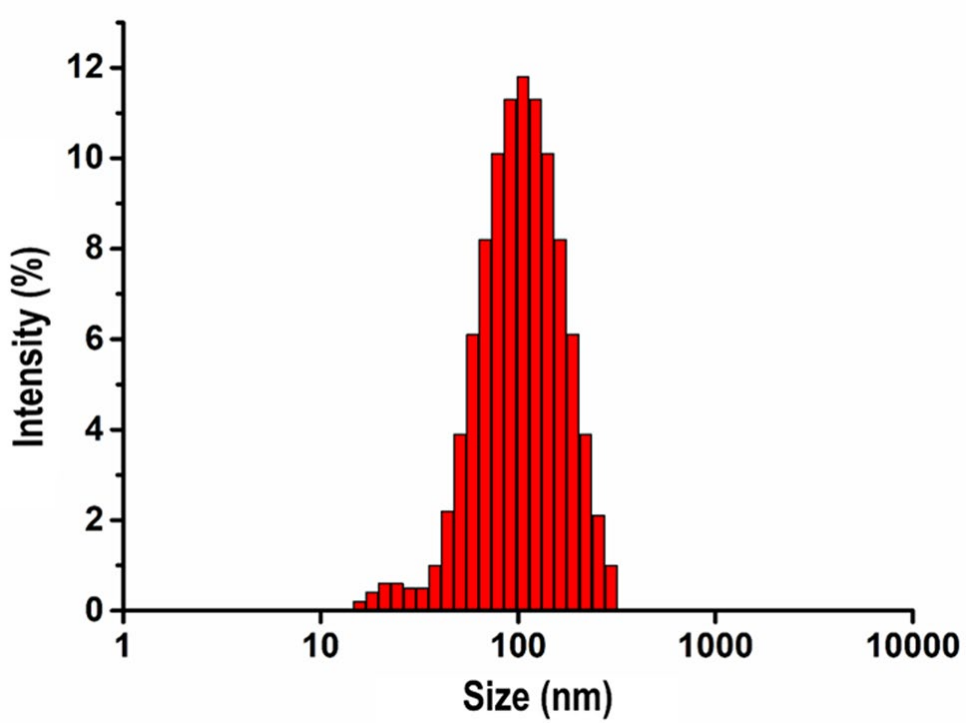
Particle size distribution graph of synthesized AgNPs

The study applied photon correlation spectroscopy to ascertain the particle size, zeta potential, and polydispersion index of AgNPs, which were derived from *S. sclarea* leaf extract. Figure 4 presents the particle size distribution, indicating that the sizes of the produced NPs vary from 15 to 295 nm, with an approximate average of 88 nm. The zeta potential was determined to be -15.8 mV.

UV-Vis spectral analysis was carried out on the S. sclarea plant extract reaction with AgNO_3_ solution, with observations taken between 300 and 600 nm at 20-, 30-, 40-, and 50-min intervals. The solution shifted in color from pale yellow to dark brown, a change associated with the formation of AgNPs. This color transformation suggests a characteristic surface plasmon resonance (SPR) feature. Peaks occurring from 400 to 467 nm are typically connected with AgNPs synthesis [40, 41, 50]. A significant SPR band emerged at 450–456 nm after 30 min, signifying the reduction of AgNO_3_ to AgNP. Hence, the synthesis was deemed optimal at 30 min.

**Fig. 5:**
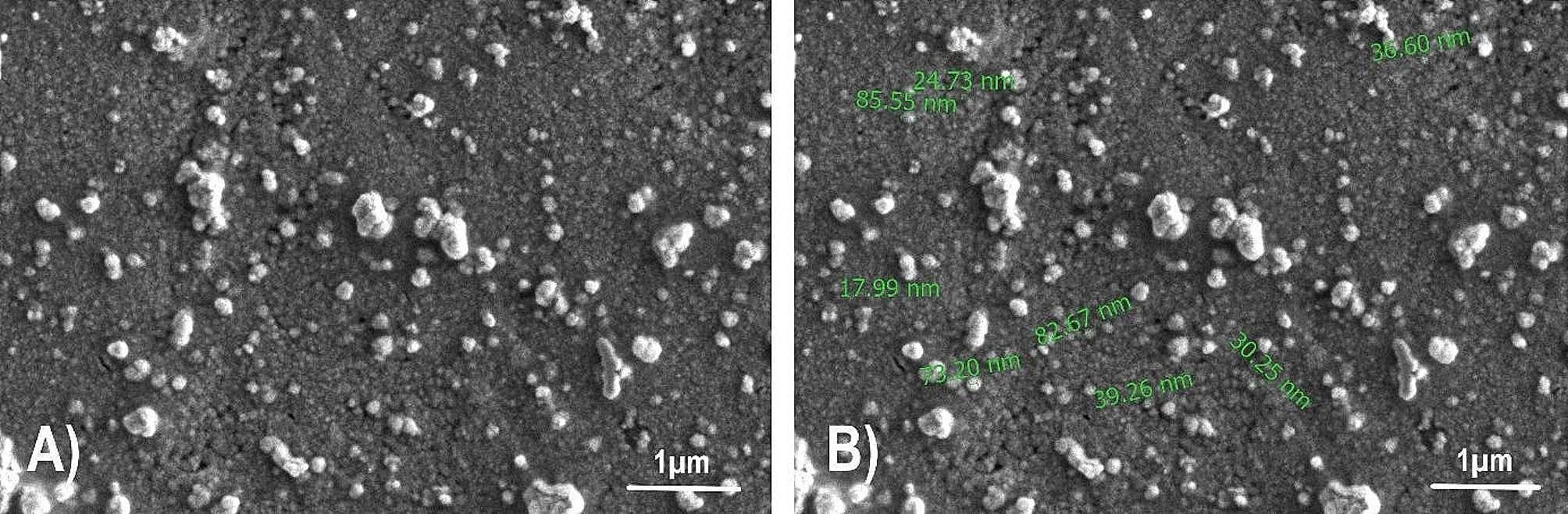
SEM images of AgNPs at 60,000× magnification under a high vacuum of 2.00 kV. The bar size is 1 μm

Morphological analyses of NPs were carried out using SEM, with a high 60,000× magnification under a 2.00 kV high vacuum. SEM images (Fig. 5) indicated the NPs were spherical and primarily exhibited a monodisperse size distribution. Additionally, the size results from the dynamic light scattering method were congruous with the NPs results. The observed characteristics of the NPs align with previous studies [51, 52].

### Morphological findings of applying AgNPs to anise callus cultures

**Fig. 6:**
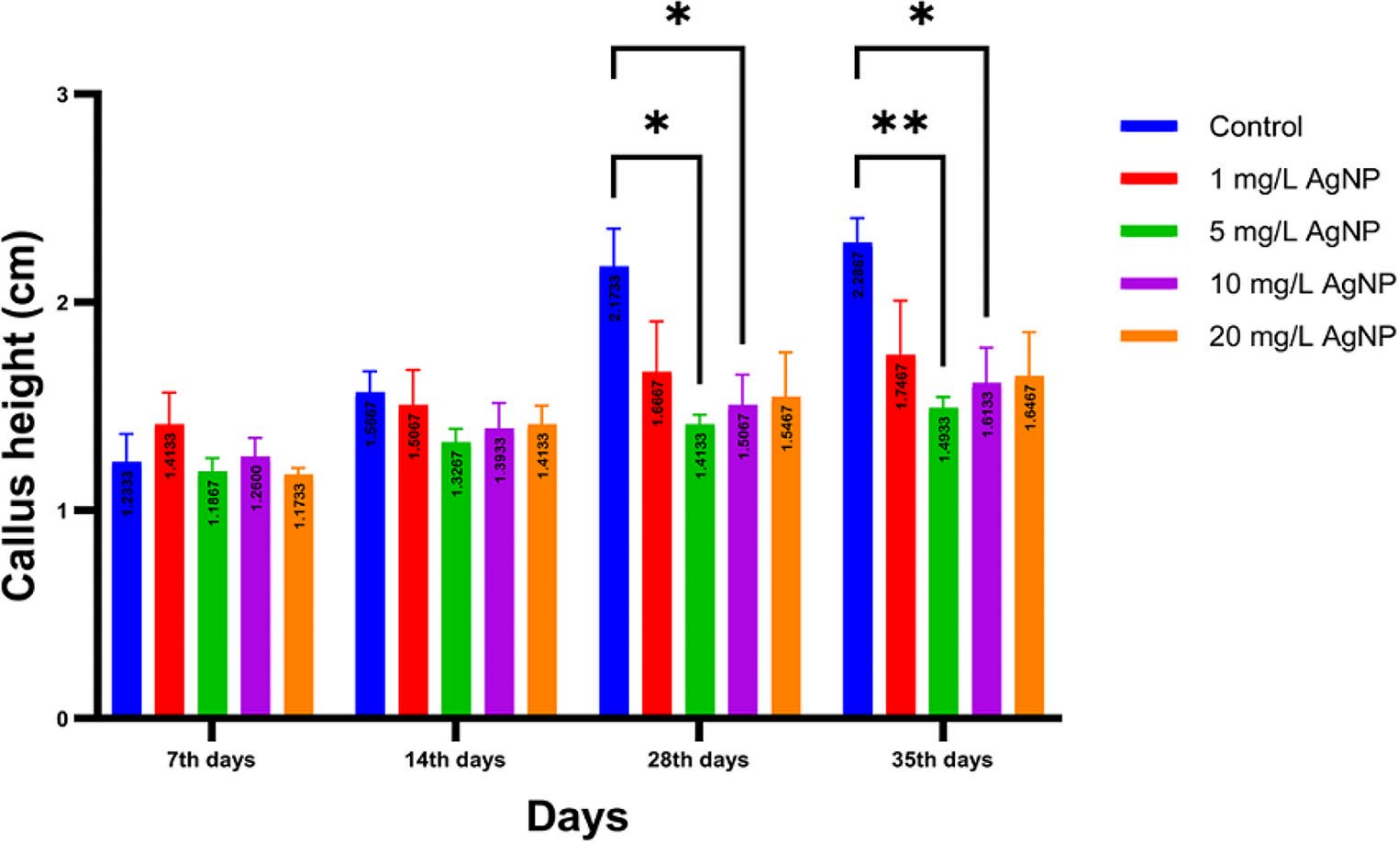
In graphs, average diameter measurements of callus developed after 7, 14, 28, and 35th days. Symbols: ^**^P<0.01 and ^*^P<0.05 mean significant difference, while “ns” means no significant difference

In all experimental sets, the callus diameter - a mass of undifferentiated plant cells - consistently expanded over time (Fig. 6). The control group registered the highest growth in diameter, ranging from 1.23 to 2.29 cm. On the seventh day, the smallest diameter (1.17 cm) was noted in the group treated with 20 mg/L AgNPs. At other time points, though, the smallest diameter (ranging from 1.32 to 1.50 cm) was recorded in the 5 mg/L AgNP-treated group. A statistically significant diameter decrease was seen on the 28th and 35th days in groups treated with 5 and 10 mg/L AgNP compared to the control group. However, this trend was not observed in any other groups. Across the board, no significant differences were noted in callus weight among different experimental sets. After the seventh day, a time-dependent decrease in growth rate occurred in all groups across callus weight.

Upon visual examination of the callus, the control group exhibited the largest diameter compared to all other groups. However, the maximum weight reduction was noted in the control group, and the group was treated with 1 mg/L. Notably, a significant diameter reduction was observed in groups treated with 5, 10, and 20 mg/L of AgNPs, yet without a corresponding significant weight loss. This implies a potential accumulation of high-molecular-weight AgNPs within plants, potentially leading to toxicity. Such accumulation explains the observed color change in the callus and MDA production, which may stem from an increase in primary and secondary fatty acid production due to cell membrane damage.

**Fig. 7:**
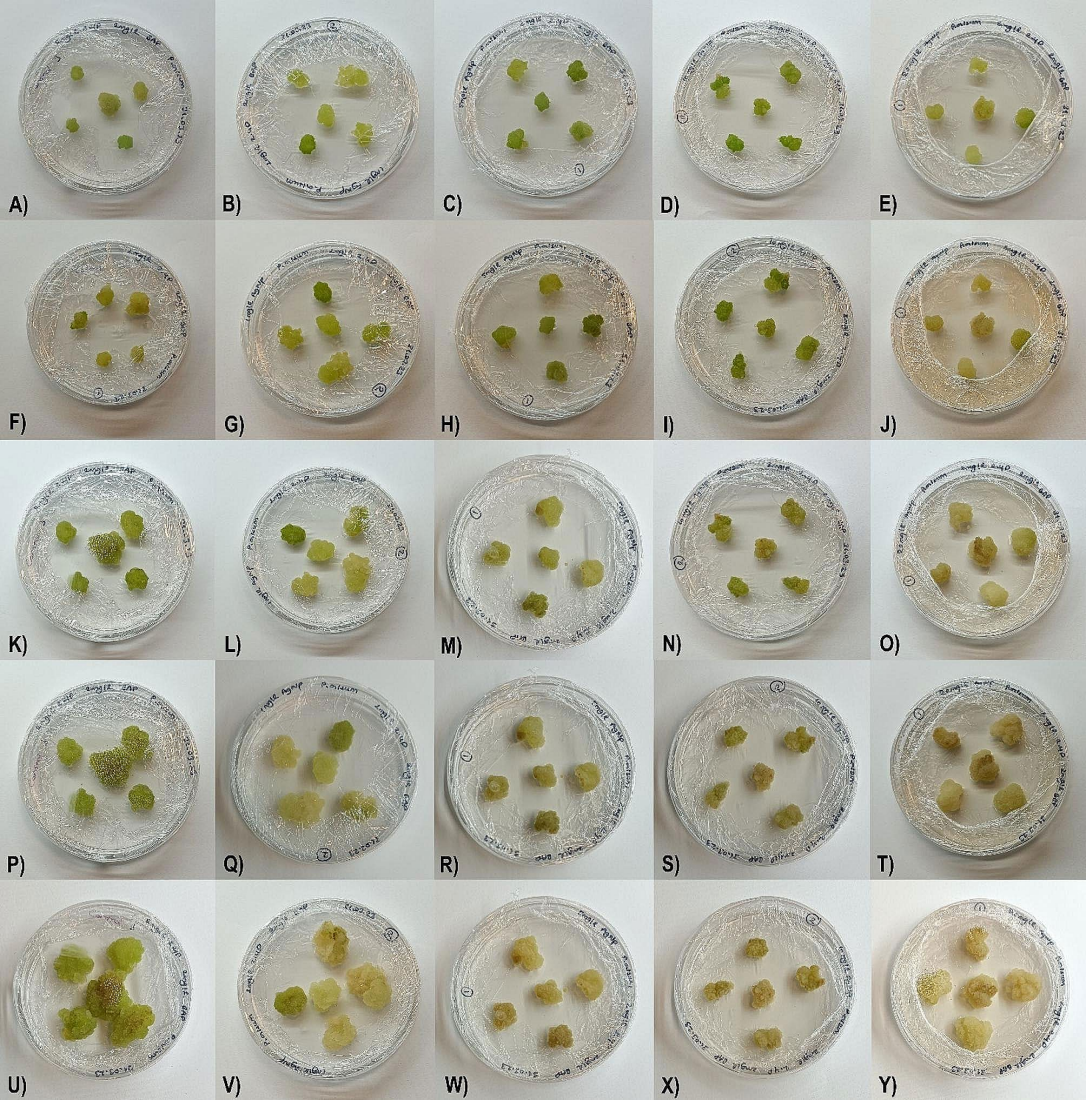
Here are the initial pictures of calli for the following experimental groups: Control group (**A**), 1 mg/L (**B**), 5 mg/L (**C**), 10 mg/L (**D**), and 20 mg/L (**E**). On the 7th day, pictures were taken of the same experimental groups: Control group (**F**), 1 mg/L (**G**), 5 mg/L (**H**), 10 mg/L (**I**), and 20 mg/L (**J**). On the 14th day, pictures were retaken: Control group (**K**), 1 mg/L (**L**), 5 mg/L (**M**), 10 mg/L (**N**), and 20 mg/L (**O**). On the 28th day, pictures were retaken: Control group (**P**), 1 mg/L (**Q**), 5 mg/L (**R**), 10 mg/L (**S**), and 20 mg/L (**T**). Finally, on the 35th day, pictures were retaken: Control group (**U**), 1 mg/L (**V**), 5 mg/L (**W**), 10 mg/L (**X**), and 20 mg/L (**Y**). It was observed that there was a significant change in the color of the callus from green to yellow or dark yellow as the concentration of AgNP increased

The study revealed that the onset of callus yellowing varied based on the tested substance’s concentration. Specifically, with a concentration of 20 mg/L, yellowing was evident on the seventh day, whereas it was marked on the 14th day for the 10 mg/L group. For the group treated with a concentration of 5 mg/L, the color transition was not observed until the 28th day, and it did not occur until the 35th day in the 1 mg/L group. Refer to Fig. 7 for a visual representation of these findings

### Toxicity levels of AgNPs in anise calli: examining tolerance and toxicity levels

**Fig. 8:**
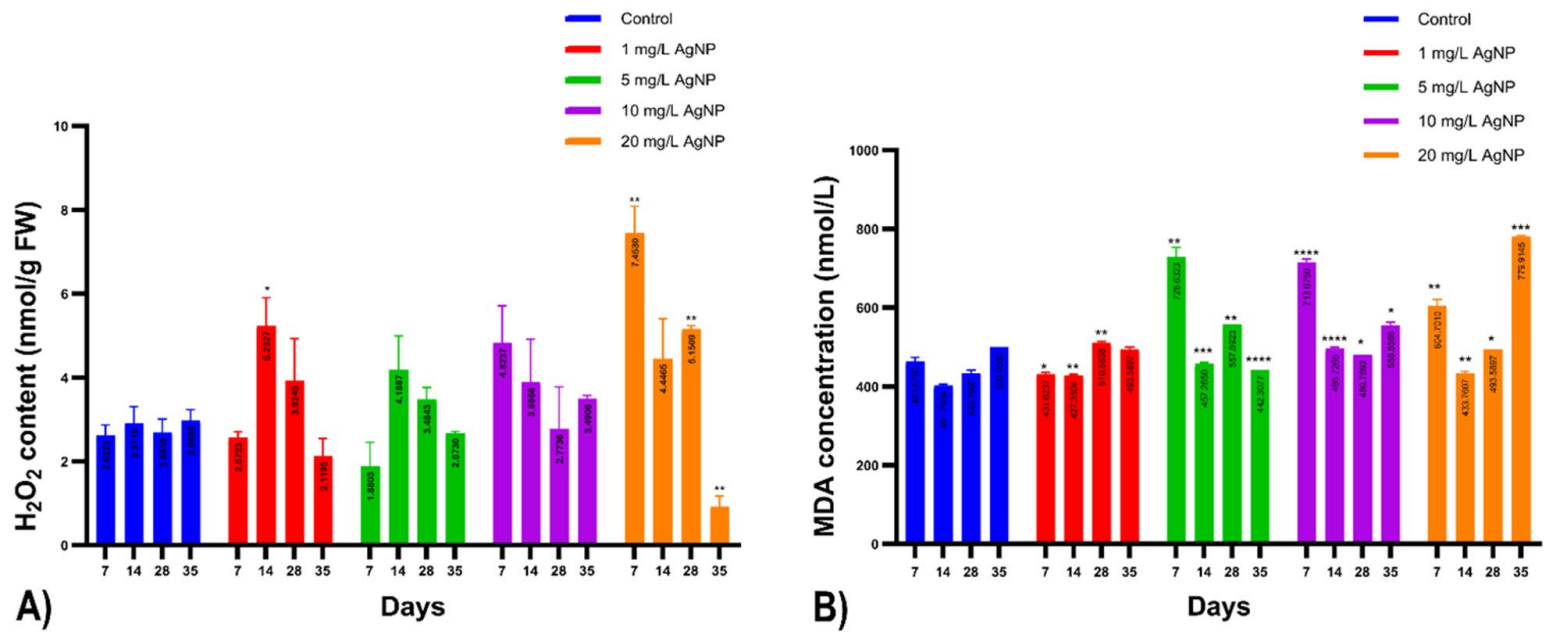
(**A**) Results of H2O2 measurements are given below, with statistical significance represented by **** *P* < 0.0001, *** *P* < 0.001, ** *P* < 0.01, and * *P* < 0.05. (**B**) The graph below shows the amount of MDA in samples treated with 1, 5, 10, and 20 mg/L of AgNP on days 7, 14, 28, and 35 compared to control samples. The statistical significance of the results is represented by **** *P* < 0.0001, *** *P* < 0.001, ** *P* < 0.01, and * *P* < 0.05

Increased ROS leads to damage to primary metabolites such as lipids, proteins, and DNA. When ROS levels surpass tolerable limits, they trigger lipid peroxidation in cell and organelle membranes, thereby disrupting normal cellular functions. By examining the behaviors of H_2_O_2_, a type of ROS, as well as antioxidant enzymes like SOD, CAT, and GPOx, which are produced to counter the harmful effects of ROS, we can better understand plant stress and toxicity [53]. The following are our research findings:

This study aimed to evaluate the activity of the H_2_O_2_ enzyme in both control and anise calli that had been treated with varying concentrations of AgNP (0, 1, 5, 10, and 20 mg/L), employing spectrophotometry. The results showed a noTable 79.70% increase on the 14th day at a concentration of 1 mg/L. The most profound effect, however, was seen on the seventh, 28th, and 35th days at a concentration of 20 mg/L. Specifically, the H_2_O_2_ level skyrocketed by 184.24% on the seventh day and 105.32% on the 28th day, whereas a substantial decrease of 69.08% was registered on the 35th day (Table 1). Even though the results were statistically significant, predominately in the 20 mg/L group, no significant variations were found in the other groups under investigation (Fig. 8-A).

**Table 1:** Change and significance levels in H_2_O_2_ measurements according to days in AgNP groups compared to the control, ** *P* < 0.01 and * *P* < 0.05 and ns (no significant difference)

	7th day	14th day	28th day	35th day
1 mg/L AgNP	1.90% ↓ ns	79.70% ↑ *	46.13% ↑ ns	26.60% ↓ ns
5 mg/L AgNP	28.29% ↓ ns	43.85% ↑ ns	29.74% ↑ ns	9.95% ↓ ns
10 mg/L AgNP	83.93% ↑ ns	33.47% ↑ ns	3.27% ↑ ns	17.58% ↑ ns
20 mg/L AgNP	184.22% ↑ **	52.71% ↑ ns	91.78% ↑ **	69.05% ↓ **

Lipid peroxidation was assessed spectrophotometrically, revealing significant differences in MDA levels across all groups except for the group exposed to 1 mg/L on the 35th day. On the seventh day, a 6.91% decrease was observed at 1 mg/L, while the 14th day saw the most significant increase – 57.15% at 5 mg/L and 53.91% at 10 mg/L. At 1 mg/L, the smallest increase was 6.38%, while the peak was 23.31% at 10 mg/L. By the 28th day, all groups showed an increase: the largest being 28.45% at 5 mg/L, and the smallest 11.15% at 10 mg/L and 13.95% at 20 mg/L. On the 35th day, a non-significant 1.45% decrease in MDA levels was found at 1 mg/L, contrasting with a notable decrease of 10.93% at 5 mg/L, as well as significant increases with 10 mg/L and 20 mg/L exposures. The most substantial increase across all groups, 55.64%, was associated with 20 mg/L AgNP exposure (Table 2).

By the seventh day, all groups, excluding the one with a 1 mg/L concentration, exhibited a notable rise in lipid peroxidation relative to the control group. This elevation persisted in the subsequent days, though it decelerated. The peak increases were noted on day 35, especially in the groups with 10 mg/L and 20 mg/L concentrations (Fig. 8-B).

**Table 2:** Changes and significance levels in the measurements of MDA, according to days in the AgNP groups compared to the control. ****P < 0.0001, ***P < 0.001, **P < 0.01, *P < 0.05 and ns

	7th day	14th day	28th day	35th day
1 mg/L AgNP	6.91% ↓ *	6.38% ↑ **	17.73% ↑ **	1.45% ↓ ns
5 mg/L AgNP	57.15% ↑ **	13.90% ↑ ***	28.45% ↑ **	10.93% ↓ ****
10 mg/L AgNP	53.91% ↑ ****	23.31% ↑ ****	11.15% ↑ *	10.92%↑ **
20 mg/L AgNP	30.40% ↑ **	8.23% ↑ **	13.95% ↑ **	55.64% ↑ ****

This study investigated antioxidant activity, particularly the activity of the GPOx enzyme in calli, measured spectrophotometrically after different AgNP concentrations (0, 1, 5, 10, and 20 mg/L) were applied. Analysis of GPOx activity revealed no significant increase between the control and 1 mg/L and 5 mg/L AgNP treatments after 7 days. However, substantial differences were evident, with a 185.43% increase at 10 mg/L and a 275.83% increase at 20 mg/L. On the 14th day, all groups showed a considerable increase, ranging from 114.60% at 1 mg/L to 332.78% at 10 mg/L. By the 28th day, there was an increase in all groups, but only 10 mg/L and 20 mg/L were statistically significant. On the 35th day, all groups had significant increases, with a modest rise at 1 mg/L and 5 mg/L, and a peak increase of 179.73% at 20 mg/L (Table 3).

**Table 3:** Change and significance levels in GPOx enzyme activity measurements according to days in AgNP groups compared to the control (**** *P* < 0.0001, *** *P* < 0.001, ** *P* < 0.01 and * *P* < 0.05 and ns)

	7th day	14th day	28th day	35th day
1 mg/L AgNP	43.83% ↑ ns	114.60% ↑ **	69.78% ↑ ns	67.57% ↑ ***
5 mg/L AgNP	30.41% ↑ ns	224.49% ↑ ***	111.34% ↑ ns	75.16% ↑ *
10 mg/L AgNP	185.43% ↑ ****	332.78% ↑ ****	174.99% ↑ *	171.35% ↑ *
20 mg/L AgNP	275.83% ↑ ****	208.87% ↑ ***	113.09% ↑ *	179.73% ↑ ***

**Fig. 9:**
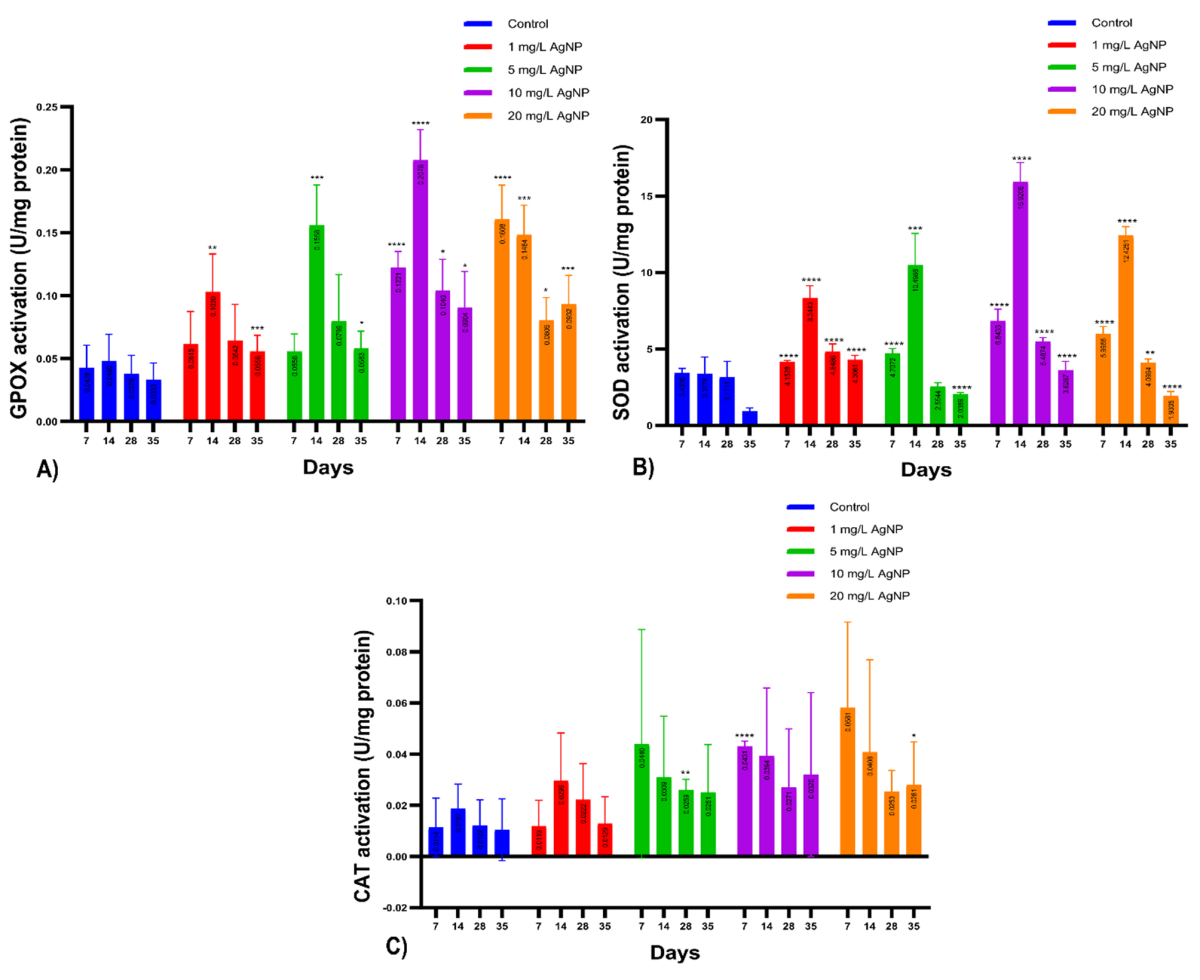
Here are the graphs for enzyme activities of GPOx, SOD, and CAT. (**A**) The first graph shows GPOx enzyme activity for 7, 14, 28, and 35 days after treatment with 1, 5, 10, and 20 mg/L AgNP and the control group. The significance levels are indicated as **** for P-value < 0.0001, *** for P-value < 0.001, ** < 0.01 and * for P-value < 0.05. (**B**) The second graph shows SOD enzyme activity for the same treatment and control groups on the same time scale. The significance levels are indicated as **** for P-value < 0.0001, *** for P-value < 0.001, and ** for P-value < 0.01. (**C**) Finally, the third graph shows CAT enzyme activity for control and 1, 5, 10, and 20 mg/L AgNP treatments on days 7, 14, 28, and 35. The significance levels are indicated as **** for P-value < 0.0001, ** for P-value < 0.01, and * for P-value < 0.05

On the seventh day, both the 10 mg/L and 20 mg/L AgNP groups demonstrated a notable rise in GPOx activity. By the fourteenth day, a significant surge was evident across all groups, particularly in the 5 mg/L, 10 mg/L, and 20 mg/L AgNP groups. On the twenty-eighth day, no significant variation was detected between the 1 mg/L and 5 mg/L groups in the absence of the 10 mg/L and 20 mg/L groups. However, by the thirty-fifth day, a marked increase was observed in all groups. These observations suggest that AgNP concentration significantly influences GPOx activity over time, with high concentrations producing a more significant effect (as depicted in Fig. 9-A).

The SOD activity evaluation results indicated notably high levels in all groups, barring the 5 mg/L group on the 28th day. On the seventh day, all groups exhibited an increase relative to the control: a modest rise of 20.94% in the 1 mg/L AgNP application group and a larger jump of 99.3% in the 10 mg/L group. By the 14th day, all groups had significantly increased, with the smallest increase of 147.04% at 1 mg/L and the most robust increase of 371.22% at 10 mg/L. Though no notable difference was found at 5 mg/L on the 28th day, SOD levels continued to rise compared to the control. Nevertheless, the rate of increase had lessened in the other groups relative to prior days. The smallest increment was observed at 20 mg/L (33.36%) and the largest at 10 mg/L (73.04%). Lastly, sizeable increases persisted in all groups on the 35th day, with the slightest increases noted at 5 mg/L and 20 mg/L and the highest increases at 1 mg/L and 10 mg/L (Table 4).

**Table 4:** Changes and significance levels of SOD enzyme activity measurements in AgNP groups compared to control (**** *P* < 0.0001, *** *P* < 0.001, ** *P* < 0.01 and * *P* < 0.05 and ns)

	7th day	14th day	28th day	35th day
1 mg/L AgNP	20.94% ↑ ****	147.04% ↑ ****	52.92% ↑ ****	335.61% ↑ ****
5 mg/L AgNP	37.10% ↑ ****	210.80% ↑ ***	19.45% ↓ ns	120.94% ↑ ****
10 mg/L AgNP	99.30% ↑ ****	371.22% ↑ ****	73.04% ↑ ****	332.14% ↑ ****
20 mg/L AgNP	74.64% ↑ ****	267.82% ↑ ****	33.36% ↑ **	118.55% ↑ ****

The study revealed that the largest uptick in SOD activity was seen in groups treated with 10 and 20 mg/L of AgNP on day 7. Additionally, a notable surge was registered in all groups on day 14, with the most prominent rise again seen in the 10 and 20 mg/L AgNP groups. By day 28, the SOD activity kept escalating in all groups, save for the 5 mg/L group, though at a reduced rate. On the 35th day, a significant surge in SOD activity was once more noted across all groups, as illustrated in Fig. 9-B.

Upon reviewing the CAT activity results, we found that the standard deviation of the CAT scores was notably higher across all groups compared to the control group. Subsequent repetitions of the experiments, thrice more, only revealed significant increases in the 10 mg/L group on day 7, the 5 mg/L group on day 28, and the 20 mg/L AgNP groups on day 35, relative to the control. This is illustrated in Fig. 9-C.

### Analysis of secondary metabolite content in anise

The calli obtained were lyophilized after 35 days. They were then subject to hexane and ethanol extractions, followed by two sessions of SM analysis utilizing a GS-MS device. The primary group in the hexane extracts was fatty acid derivatives. The main components identified in all extracts were palmitic acid TMS derivative, 9,12-Octadecadienoic acid (Linoleic acid) (Z, Z)-TMS derivative, and α-Linolenic acid TMS derivative. In the control group’s hexane extract, fatty acid derivative components accounted for 86.3%. This proportion increased to between 92.7 and 96.4% in hexane extracts treated with AgNP. Thus, there was a noticeable uptick in total fatty acid derivative components in the experimental groups compared to the control group. Anethole, a principal active secondary ingredient among phenolic compounds, was absent in both the control and 10 mg/L groups, with only minimal amounts in the experimental groups. This study confirmed, for the first time, the existence of vanillyl alcohol, an essential phenolic compound with anti-inflammatory properties (Table 5).

**Table 5:** The chemical composition of hexane extracts in *P. anisum* callus cultures exposed to AgNPs.

RT	RRI	Compound	Control (%)	1 mg/L AgNPs (%)	5 mg/L AgNPs (%)	10 mg/L AgNPs (%)	20 mg/L AgNPs (%)
		**Fatty Acid Derivatives**					
28.568	1496	Octadecanoic acid, TMS derivative	-	tr	0.1	0.1	0.1
40.331	1890	Dodecanoic acid (Lauric acid), TMS derivative	-	-	-	0.1	0.3
45.782	2108	Tetradecanoic acid (Myristic acid), TMS derivative	0.4	0.4	0.1	0.5	0.6
48.687	2233	Pentadecanoic acid (Pentadecylic acid), TMS derivative	1.1	1.1	0.8	1.3	1.6
53.200	2422	Hexadecanoic acid (Palmitic acid), TMS derivative	30.2	28.6	23.4	39.9	48.8
54.182	2457	Heptadecanoic acid (Margaric acid), TMS derivative	0.4	1.6	4.7	1.1	1.8
59.078	2606	9-Octadecanoic acid (Oleic acid, (*Z*)-, TMS derivative	1.8	-	1.9	5.5	-
59.304	2612	Stearic acid, TMS derivative	-	--	-	-	8.9
61.103	2655	Cis- 9,12-Octadecadienoic acid (α-Linoleic acid) (*Z*, *Z*)- TMS derivative	35.2	41.4	32.9	33.0	19.1
62.654	2692	α- Linolenic acid, TMS derivative	16.3	22.2	31.8	12.4	6.2
66.249	2770	11-Eicosenoic acid (*E*)-, TMS derivative	0.9	-	-	-	-
68.995	2826	Arachidic acid, TMS derivative	-	-	-	1.0	2.5
83.051	3088	Behenic acid (docosanoic acid), TMS derivative	-	0.3	0.7	1.3	2.8
		***n*-alkane derivative** (**Volatile oil**)					
28.856	1504	Pentadecan	-	tr	tr	-	0.1
		**Phenolic content**					
29.369	1520	Vanillyl alcohol, 2TMS derivative	2.2	1.8	0.7	2.7	3.8
39.565	1862	t-Anethole	-	tr	0.1	-	0.1
		**Carbohydrate derivatives**					
38.022	1805	D-(-)- Tagatofuranose, pentakis (trimethylsilyl) ether (isomer 1)	1.0	-	-	-	-
38.485	1822	D-(-)- Tagatofuranose, pentakis (trimethylsilyl) ether (isomer 2)	2.9	-	-	-	-
		**TOTAL**	**92.4**	**97.4**	**97.2**	**98.9**	**96.7**

RT: Retention time; RRI: Relative retention time

Our study found that carbohydrate derivatives, which are the primary metabolites in the ethanol extract of the control group, made up the largest group (69.2%). The primary components of these derivatives were D-(-)-Tagatofuranose, pentakis(trimethylsilyl) ether (isomer 2), and β-D-Glucopyranose. However, when the concentration of AgNP was increased to 5 mg/L, 10 mg/L, or 20 mg/L, there was a pronounced decrease in carbohydrate derivatives and a corresponding rise in fatty acid derivatives. Phenolic compounds, including vanillyl alcohol and 4-Hydroxybenzoic acid (known for their antimicrobial properties), were also detected in the ethanol extract. It is noteworthy that the concentration of these compounds increased significantly in direct proportion to the amount of AgNP used (Table 6).

**Table 6:** The chemical composition of ethanol extracts in *P. anisum* callus cultures exposed to AgNPs

RT	RRI	Compound	Control (%)	1 mg/L AgNPs (%)	5 mg/L AgNPs (%)	10 mg/L AgNPs (%)	20 mg/L AgNPs (%)
		**Fatty Acid Derivatives**					
28.636	1497	Octadecanoic acid, TMS derivative	-	-	0.1	0.1	0.1
33.664	1655	Butanedioic acid (Succinic acid), 2TMS derivative	-	-	1.0	1.6	1.5
34.133	1670	2-Butenedioic acid (Fumaric acid), (*E*)-, 2TMS derivative	-	-	0.2	-	-
37.634	1791	Pentanedioic Acid (Glutaric Acid), 2TMS derivative	-	-	tr	-	-
45.906	2113	Tetradecanoic acid (Myristic acid), TMS derivative	-	-	0.2	0.4	0.4
48.646	2231	Pentadecanoic acid (Pentadecylic acid), TMS derivative	-	-	1.0	0.8	0.7
49.910	2288	Hexadecanoic acid, ethyl ester	1.0	-	-	-	-
51.306	2347	Heptadecanoic acid, ethyl ester	-	2.3	-	-	1.3
52.702	2405	Hexadecanoic acid (Palmitic acid), TMS derivative	-	-	40.1	40.4	38.8
54.354	2464	Heptadecanoic acid (Margaric acid), TMS derivative	-	-	1.8	0.8	1.0
58.410	2588	9-Octadecanoic acid (Oleic acid, (*Z*)-, TMS derivative	-	-	-	5.0	5.4
58.914	2602	Stearic acid, TMS derivative	-	-	4.3	-	-
59.594	2619	Tridecanoic acid (Tridecylic acid)	-	41.9	-	-	-
61.627	2667	Cis-9,12-Octadecadienoic acid (α-Linoleic acid) (*Z*, *Z*)- TMS derivative	-	-	32.8	17.8	4.6
62.706	2693	α- Linolenic acid, TMS derivative	-	-	6.1	5.5	1.1
66.679	2779	11-Eicosenoic acid	15.6	-	-	-	-
83.018	3087	trans-9,12-Octadecadienoic acid, (Linoelaidic acid)	7.6	-	-	-	-
		***n-alkane*derivative** (**Volatile oil**)					
29.011	1509	Pentadecan	-	-	0.1	0.3	0.8
33.079	1635	Hexadecane (cetane)	0.7	-	-	-	-
35.418	1713	Heptadecane	-	-	0.1	0.3	0.8
		**Phenolic content**					
29.369	1520	Vanillyl alcohol, 2TMS derivative	-	2.2	1.4	6.8	10.9
40.490	1896	Salicylic acid (2-hydroxybenzoic acid-aspirin), 2TMS derivative	-	-	0.6	-	-
44.510	2055	4-Hydroxybenzoic acid (4-carboxy phenol), 2TMS derivative	-	-	1.3	1.3	3.7
40.276	1888	Thiamazole (methimazole), 2TMS derivative	-	-	0.2	-	-
		**Carbohydrate derivatives**					
36.768	1760	β-D-Ksilopiranoz, 4TMS derivative	3.0	2.5	-	-	-
36.985	1768	β-Galaktofuranoz, TMS derivative	9.5	-	-	-	-
37.665	1792	D-(-)- Tagatofuranose, pentakis (trimethylsilyl) ether (isomer 1)	-	6.1	0.9	6.7	4.4
38.05	1806	D-(-)- Tagatofuranose, pentakis (trimethylsilyl) ether (isomer 2)	26.2	24.9	0.4	1.1	11.6
39.644	1865	β-D-Mannopyranose, 5TMS derivative	11.1	5.9	-	-	-
42.997	1994	β-D-Glukopiranoz, 5TMS derivative	19.4	8.9	-	-	-
		**TOTAL**	**94.1**	**94.7**	**92.6**	**88.9**	**87.1**

RT: Retention time; RRI: Relative retention time

## Discussion

Numerous studies have explored the influence of AgNPs on plant morphology. In a survey of chrysanthemum plants, it was noted that an increase in AgNP concentration led to a reduction in the diameter of both callus and adventitious roots compared to the control group. Further, a color shift from green callus to brownish was noted after the third week [54]. In another study examining the effect of various AgNP concentrations on sugarcane callus, calli exposed to AgNPs manifested shorter and more swollen characteristics, a change more prominent at higher concentrations [55].

On examining callus morphology, the control group registered the highest diameter amongst all groups. Despite a marked reduction in diameter being observed, particularly in groups treated with 5, 10, and 20 mg/L of AgNPs, no significant weight loss was seen. The greatest weight decrease was recorded in the control group and the group treated with 1 mg/L of AgNPs. This indicates that AgNP stress arising from accumulation can trigger a change in the callus’ color and Malondialdehyde (MDA) production. Any increase in primary and secondary fatty acid production due to cell membrane damage might prompt these color changes [56]. Hence, our findings suggest that plants can accumulate high-molecular-weight AgNPs, potentially leading to toxicity.

H_2_O_2_ is a byproduct of respiration, contributing to physiological regulation at minimal levels; however, its high concentrations can lead to cell death. It encourages stress tolerance when present in low quantities but impedes cell functions in abundance. A study by Nair and Chung (2014b) revealed a notable increase in H_2_O_2_ levels in the roots and stems of *Oryza sativa* upon exposing them to different AgNPs concentrations (0.2, 0.5, and 1 mg/L).

Similarly, Cvjetko et al. [57] carried out a study on *Nicotiana tabacum* to examine the impact of various AgNP concentrations (25, 50, 75, 100, and 500 µM) on ROS located in roots and leaves. Their findings showed that all tested AgNP concentrations raised ROS levels in roots compared to the control, while in the leaves, ROS levels decreased as the AgNP concentration increased.

Furthermore, Yanık and Vardar (2019) confirmed a dose-dependent increase in H_2_O_2_ in wheat roots caused by different amounts of AgNP. Scavenging activities manage the presence of H_2_O_2_ inside cells. An overflow of H_2_O_2_ levels beyond a particular limit could trigger adaptation responses to AgNP. Alterations in cytosolic H_2_O_2_ levels could result from changes in plasma membrane gradients or mitochondrial activity under stress [58].

In our study, we identified a fluctuation in H_2_O_2_ levels depending on the dosage and duration. We saw a substantial increase at 1 mg/L on the 14th day and 20 mg/mL on the seventh and 28th days. However, a significant decrease was noted at 20 mg/L on the 35th day.

When the quantity of ROS in a cell surpasses its antioxidant defenses, lipid peroxidation increases, disrupting the cell’s regular physiological processes. MDA, a common byproduct produced from lipid peroxidation, can damage cell membranes, reduce fluidity and ion transport, and cause protein cross-linking and loss of enzyme activity [59]. Research has shown that varying concentrations of AgNP increase MDA levels in *O. sativa*, *A. thaliana*, *V. radiata*, *P. sativum*, and *N. tabacum* [57, 60–62]. Similarly, a study by Yanık and Vardar [63] found that AgNPs resulted in MDA accumulation in wheat roots. Our study also found varying amounts of MDA based on both concentration and number of days. On the seventh day, MDA levels were notably higher in all experimental groups, except the 1 mg/L group, showing a significant rise compared to the control group. This elevated level persisted throughout subsequent days, with a notable peak on the seventh and 35th days, particularly at concentrations of 10 mg/L and 20 mg/L, albeit at a decreasing rate.

During lipid peroxidation, a series of reactions occur, resulting in the creation of termination products. These primarily include aldehydes such as propenal, hexanal, 4-hydroxynonenal, and lipid hydroperoxides [44, 59, 64]. The type of byproducts formed varies based on the intensity and duration of stress. Lower MDA levels do not always signify reduced lipid peroxidation, as alternative products may be produced. In our study, significant stress induced by AgNP was observed in all groups except the 1 mg/L group, notably on the seventh day. Despite the plant’s attempt to adapt in the subsequent days, the stress level rose in line with the H_2_O_2_ results, especially at 20 mg/L after the 28th day. This variation suggests toxicity, particularly at 20 mg/L, and is correlated with an increase in fatty acids and degradation of the cell membrane.

Several studies suggest that the activity and gene expression of the SOD enzyme in *Ricinus communis*, *A .thaliana*, *V. radiata*, and *P. sativum* is enhanced by AgNPs [34, 62, 65]. However, a different report indicates that concentrations of 40 and 80 mg/L AgNP led to reduced SOD activity in *Solanum lycopersicum* leaves [66]. Another research showed, the only exception being a 50 µM group, an overall decrease in SOD activity in *N. tabacum* roots after AgNPs application [57]. Yanık and Vardar (2019) attribute this variation to dosage, stating SOD increases at lower AgNP doses but decreases at higher doses. In this study’s SOD analysis, an increased activity across all groups and days was noted. Particularly, all groups exhibited a significant rise on the 14th day, with the 10 and 20 mg/L AgNP groups showing the greatest increase.

Many studies have analyzed the impact of AgNPs on GPOx activity in plants. Yasur and Rani [67] found an increase in GPOx activity in *R. communis* seeds exposed to AgNP concentrations between 500 and 4000 mg/L. Similarly, Kaveh et al. [34] deduced that Peroxidase-linked gene expressions increased in *A. thaliana* exposed to 5 mg/L AgNP. Conversely, Cvjetko et al. [57] noted a decline in pyrogallol peroxidase activity in *N. tabacum* roots and leaves due to AgNP. Yanık and Vardar [63] witnessed fluctuation in GPOx activity when exposed to varying AgNP concentrations, with a notable rise at 0.5 ppm compared to the control.

Our study observed comparable variable increases in GPOx activity, contingent on the dose and exposure length to AgNP. We noted the highest escalation in GPOx activity on the seventh day in groups administered with 10 and 20 mg/L AgNP. However, on the fourteenth day, the highest spike was exhibited across all groups, showing similar toxicity to the SOD enzyme. The GPOx activity continued to rise in the following days, albeit at a diminishing pace.

Increased CAT activity and expression of CAT-related genes in various plants treated with AgNPs have been identified in several studies [57, 60, 61, 67]. Conversely, Cekic et al. [66] documented a drop in CAT activity in AgNPs-treated *S. lycopersicum* leaves at concentrations of 20, 40, 60, and 80 mg/L. Another investigation saw no notable changes in *Lemna minor*’s CAT activity when exposed to 0.05 and 2 mg/L of AgNPs, although there was a decrease in their growth rates and leaf count [68]. In our study, we noticed analogous results. AgNPs appeared to have little impact on CAT activity, as negligible differences were noted across nearly all groups. Thus, we can conclude that CAT activity largely remains constant.

The study results reveal that 1, 5, and 10 mg/L applications of AgNP provide active antioxidant activity. Yet, an increase in the dosage to a 20 mg/L concentration impairs the plant’s defense mechanism. Evidently, the plant can withstand a dose of 1 mg/L AgNP due to its high tolerance, even when toxicity levels increase at 5 mg/L. Unfortunately, at 10 and 20 mg/L, toxicity levels rise significantly, with the 20 mg/L dosage severely limiting plant functionality.

Evidence suggests that alterations in fatty acid levels can destabilize cells’ redox state, increasing ROS production and reducing antioxidant enzyme activity [69]. Therefore, fatty acids might control oxidative stress physiologically [64]. Our findings indicate a possible correlation between increased fatty acid and antioxidant enzyme levels and balanced oxidative stress.

Plants leverage carbohydrates, chiefly generated from photosynthesis, for energy and to manufacture compounds like cellulose and starch. Cellulose forms the cell wall, while starch, stored in seeds and other plant regions, acts as a food source. Our current study performed SM analyses on 35-day calli, revealing that carbohydrate derivatives made up 69.2% of the ethanol extract in a control group. Primary components of 5TMS derivatives were identified as D-(-)-Tagatofuranose, Pentakis (trimethylsilyl) ether (Isomer 2), and β-D-Glucopyranose. A noted decrease in carbohydrate derivatives and a subsequent increase in fatty acid derivatives occurred in concentrations of 5 mg/L, 10 mg/L, and 20 mg/L of AgNP. Fatty acids form the phospholipid bilayer; ensuring their synthesis is crucial for maintaining membrane functions [70, 71]. Spikes in fatty acid levels imply membrane remodeling or synthesis when adapting to adverse conditions [72]. Fatty acid derivatives accounted for a significant portion of the hexane extracts in this study. The main components in all extracts included Saturated Fatty Acid (SFA) Palmitic acid TMS, Polyunsaturated Fatty Acid (PUFA) 9,12-Octadecadienoic acid (Linoleic acid)(Z, Z)-TMS, and a Monounsaturated Fatty Acid (UFA) α-Linolenic acid TMS derivative. The control group’s hexane extract displayed 86.3% fatty acid derivative components, while 92.7-96.4% was observed in the hexane extracts of the AgNP-treated groups. This increase points to a potential degradation of the plant cell membrane. Kaveh et al. [34] have cited that AgNPs stimulate certain genes associated with plant defense against insects, pathogens (AT1G52040), and wounds (AT2G01520).

In the literature, Rebey et al. [16] analyzed anise seeds from four countries based on ripening times. They found the ripe seeds contained the most fatty acids. Other past studies have supported the notion that anise seeds are lipid-rich and that fatty acids make up 5–11% of their total mass [19, 73, 74]. However, one study found that water stress led to a notable dip in the total fatty acid content of aniseed [75]. Our study observed a significant rise in SFA, UFA, and PUFA content levels in food, particularly at 1 and 5 mg/L concentrations.

PUFAs and unsaturated fatty acids (UFAs) are crucial for lowering cholesterol levels and enhancing cardiovascular health, making them key components of functional foods and dietary supplements [76, 77]. Linoleic acid, one of the primary forms of PUFAs, is the second most abundant type found in anise seed oil. This essential fatty acid supports human health in numerous ways, including maintaining membrane stability and controlling inflammation [78, 79]. Previous studies reported that linoleic acid constituted 20% of nearly mature anise seeds [16]. However, the current study detected an approximately two-fold increase at 1 and 5 mg/L concentrations within a significantly shorter time frame.

Plants adjust their membrane lipids in response to environmental stress. It is believed that free radical reactions break down these lipids, with oxidative stress primarily affecting the membranes. This can result in a decrease in lipid content and an inhibition of lipid biosynthesis [80]. Stress-acclimated plants largely regulate the fluidity of their membrane lipids by altering their PUFA content. This regulation aids the functioning of crucial, integral proteins, enabling the plant to tackle various stress types [70]. Thus, controlling fatty acid synthesis serves as an adaptive mechanism for plants to shield their membranes from oxidative stress. This study demonstrates significant changes in fatty acid composition, potentially leading to a rise in lipid MDA peroxidation content, a marker of oxidative lipid damage.

Vanillyl alcohol, a pivotal phenolic compound found in food and perfumes, is renowned for its enticing aroma and utilization as an intermediate in drug and chemical synthesis [81]. Moreover, it has been found to possess anti-inflammatory properties and function as a crucial antioxidant [82]. Our study marks the first identification of vanillyl alcohol, noting an increase in its production, particularly in applications of 10 and 20 mg/L of AgNP. Similarly, this is the first study to report the identification of 4-Hydroxybenzoic acid. Previous research has demonstrated this phenolic’s antibacterial, antifungal, antiviral, and antioxidant capabilities [83, 84]. In our study, 4-Hydroxybenzoic acid was found in both 5 and 10 mg/L groups, with its highest concentration present in the 20 mg/L group.

SMs such as phenols and salicylic acid play a key role in plant development and tolerance mechanisms, particularly during conditions of biotic and abiotic stress. These phenolic compounds are created through the shikimate/phenylpropanoid or polyketide acetate/malonate pathway, where they produce both monomeric and polymeric phenolics. These entities significantly contribute to stress prevention and physiological regulation. They also govern defense mechanisms both above and below ground and orchestrate the synthesis of thousands of phenolic compounds for survival in fluctuating environments. Such environments often involve factors like high light intensity, cold, drought, and heavy metal exposure, which prompt an increase in phenolic accumulation to counteract any potential toxicity [85]. To summarize, these compounds not only fortify the plant against stressors but also regulate its various functions. Some polyphenols even serve as scavengers of ROS and act as protective agents.

The green synthesis of AgNPs offers a practical and eco-friendly alternative for bulk production. It is favored over other biosynthetic methods due to its efficiency and effectiveness. Various studies suggest that plant extracts combined with AgNPs have a more synergistic effect against microbial strains and cancer cell lines compared to the use of plant extracts alone [86].

The utilization of extracts from diverse medicinal plants underscores the potential for the green synthesis of AgNPs. Notably, different plant species lend different properties to the NPs. For example, *Solanum tuberosum*, *Phyllanthus niruri*, and *Calotropis gigantea*-derived NPs possess antibacterial qualities. Antifungal properties are found in NPs synthesized from green and black tea leaf extract and *Chlorella vulgaris*, *Scindapsus officinalis*, *Pandanus odorifer* Leaf, aqueous pine park extracts, and *Citrus maxima* plant-derived NPs show anticancer properties [87].

In addition to these benefits, studies show that biosynthesized NPs have protective effects on the liver, kidney, and body weight in animal models, adding to their potential clinical applicability [88]. This indicates that AgNPs not only possess these properties but may also offer therapeutic benefits for other physiological concerns.

This study examines the effectiveness of green-synthesized AgNPs in anise, a medicinal plant, providing a significant addition to existing research. This comparatively unexplored area could offer substantial insights into the potential use of green-synthesized AgNPs to enhance the medicinal properties of anise.

## Conclusions

Our study found that applying AgNPs increases the fatty acid content and disrupts cell membranes in anise calli. We identified small quantities of trans-anethole in the AgNP applications, emphasizing the need to ascertain the production of vanillyl alcohol and 4-hydroxybenzoic acids. The plant encountered stress during the initial 14 days but later adapted to its environment. Applying 10 and 20 mg/L of AgNP led to an increase in toxic values, while PUFA and UFAs notably rose in the 1 and 5 mg/L AgNP groups. Importantly, our research demonstrated that AgNP stress influences the fatty acid content in anise calli, causing a surge in key SMs such as palmitic and linoleic acid. PUFA and UFAs also significantly increased in some AgNP groups, proving crucial to the pharmaceutical industry. Notably, in vitro, callus cultures can produce valuable metabolites more quickly. Future research should examine the impact of AgNP treatments on anise SMs levels during early stages to better understand the potential of AgNPs in generating medically significant anise SMs. Lastly, studying the effects of green-synthesized AgNPs on various plant species could yield significant insights into health, agriculture, and the food industry.

## Data Availability

All data collected, observed, or generated during the course of our research will be considered as research data. This includes numerical data, observations, experimental results, and any other relevant information that can be used to support or verify research findings. These data may include digital records, text documents, and images, which are essential sources of information in scientific research. The project coordinator will store all data, including enzymatic and non-enzymatic antioxidant data, statistical calculations, and files such as Word, Excel, PNG, TIFF, and JPG in data banks for future reference.
